# Highly Adhesive and Sustainable UV/Heat Dual-Curable Adhesives Embedded with Reactive Core-Shell Polymer Nanoparticles for Super-Narrow Bezel Display

**DOI:** 10.3390/ma13163492

**Published:** 2020-08-07

**Authors:** Jun Hyup Lee

**Affiliations:** Department of Chemical Engineering, Soongsil University, Seoul 06978, Korea; junhyuplee@ssu.ac.kr; Tel.: +82-2-829-8329

**Keywords:** adhesion strength, display adhesive, liquid crystal display, polymer nanoparticle, super-narrow bezel

## Abstract

To achieve the seamless characteristics of displays, liquid crystal (LC) devices need a super-narrow bezel design. This device architecture can be constructed using functional adhesives that possess excellent physical and chemical properties. In this study, mechanically robust ultraviolet (UV)/heat dual-curable adhesives with outstanding reliability and processability have been fabricated using reactive poly(methyl methacrylate) (PMMA)/polyethyleneimine (PEI) core-shell nanoparticles. Their curing characteristics, narrow drawing processability, adhesive strength, elongation at break, and the contact contamination of LCs have been investigated. Compared to conventional adhesive material, the proposed adhesive containing multifunctional PMMA/PEI nanoparticles afforded a high adhesion strength of 40.2 kgf cm^−2^ and a high elongation of 64.8% due to the formation of a firm crosslinked network with matrix resins comprising bisphenol A epoxy resin and bisphenol A glycerolate dimethacrylate. Moreover, the proposed adhesive showed an excellent narrow drawing width of 1.2 mm, which is a prerequisite for super-narrow bezel display. With regard to LC contamination, it was found that the level of contamination could be remarkably reduced to 61 µm by a high-temperature curing process. This study makes a significant contribution to the development of advanced display, because it provides robust and sustainable display adhesives based on nanomaterials, thereby enhancing the life and sustained operability of displays.

## 1. Introduction

Liquid crystal (LC) displays have been widely used in large-sized applications, such as tablets, monitors, televisions, and digital signage panels. In modern manufacturing technology for large-area displays, the display bezel, which is the outer frame around the active screen area, is getting thinner to facilitate larger display screens and provide a truly immersive experience for users. For this reason, display adhesive in the narrow bezel area of a device requires effective properties, such as high adhesive strength, high toughness, narrow wettability, and low LC contact pollution. The previous studies for increasing the adhesive strength have utilized various methods, such as polyblending or curing with polythiourethanes and epoxy-acrylate oligomers [1,2,3,4]. Polyblending the epoxy resin with lignin can promote the curing degree and shear strength of epoxy adhesives [1,2]. Polythiourethanes can be used as polymeric hardeners for increasing the adhesive properties and promoting the low-temperature curing reaction [3]. Some researchers have synthesized partially acrylated resin based on epoxy-acrylate oligomers for dual curing and have investigated their mechanical properties [4].

Commonly, epoxy resin shows several desirable properties, such as high chemical resistance and excellent adhesive properties [5,6,7]. Hence, epoxy resins are widely used in protective coatings and electrical laminates as well as in adhesives. In particular, adhesive materials based on epoxy resins are highly suitable for bonding with various surfaces, such as steel, plastic, wood, and composites owing to the formation of a network-like structure after curing of the epoxy resin [8]. However, epoxy resin, which is a thermosetting polymer, is typically brittle owing to its high crosslinking density. As a consequence, it shows low impact resistance and is prone to formation of cracks. For this reason, tougheners are commonly employed in most commercial adhesives containing epoxy resins. Usually, carboxyl-terminated butadiene acrylonitrile copolymers (CTBNs), amine-terminated butadiene acrylonitrile copolymers (ATBNs), and epoxy-terminated butadiene acrylonitrile copolymers (ETBNs) are used as tougheners for epoxy resins [9,10]. However, the isolated rubber particles are formed due to phase separation during the curing process, and such phase separation can adversely affect the fracture toughness of modified epoxies. Moreover, it is found to decrease the thermal and mechanical properties owing to some part of the rubber remaining in the epoxy continuous phase. In addition, CTBNs may reduce the glass transition temperature (T_g_) of the epoxy matrix.

Recently, nano-sized filler particles can be used as a candidate for toughener of epoxy resin in order to overcome these undesirable side effects [11,12,13,14,15,16,17]. A filler accounting for most of the adhesive material is usually an inorganic material, such as silicon dioxide, calcium carbonate, magnesium carbonate, magnesium sulfate, aluminum oxide (alumina), magnesium oxide, iron oxide, and glass fiber. The interfacial morphology of the filler greatly affects its properties. The mechanical properties, such as tensile strength, are reduced by an increase in the particle size due to low surface area of filler particles [11,12]. In addition, an increase in filler content and specific surface area serves to increase the mechanical properties of the adhesive [18]. Especially, core-shell particles (CSPs), which consist of soft rubbery cores and hard shells around them, are used as tougheners for epoxy resin, and they are well-dispersed within the epoxy system [16,17]. Moreover, CSPs have the advantage of easily being able to control their particle size.

**Figure 1:**
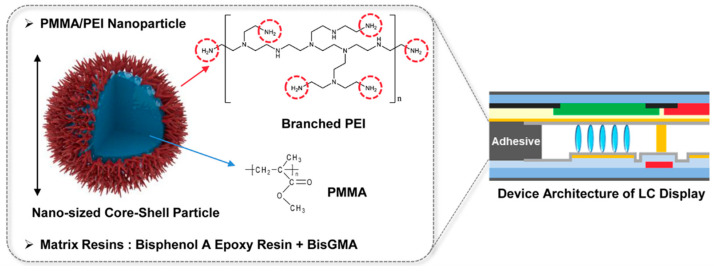
Schematic diagram of UV/heat dual-curable adhesive embedded with reactive core-shell polymer nanoparticles for super-narrow bezel display.

In this work, highly adhesive and sustainable UV/heat dual-curable adhesives with high toughness and low LC contamination level for super-narrow bezel display have been prepared using reactive core-shell polymer nanoparticles. As shown in Figure 1, a functional polymer nanoparticle comprising poly(methyl methacrylate) (PMMA) as a core and polyethyleneimine (PEI) shell was prepared through one-pot graft copolymerization. The UV/heat dual curable adhesive embedded with reactive PMMA/PEI polymer nanoparticles was fabricated with reactive resins comprising bisphenol A epoxy resin and bisphenol A glycerolate dimethacrylate (BisGMA) by using revolution–rotation mixing process. The embedded core-shell polymer nanoparticles with a number of amine reactive groups play a dual function in the toughening and multi-curing of epoxy resin within the adhesive through formation of a crosslinked nanoparticle network, so that the adhesive can possess high adhesion strength, high toughness, and low LC contamination. In addition, the proposed adhesive can be drawn narrowly due to nano-sized nature of core-shell polymer nanoparticle. It is expected that the proposed adhesive materials, which have the advantages of excellent mechanical properties, narrow processability, and outstanding reliability, could be useful in various electronic fields, such as advanced optical devices, sensors, and flexible displays. The adhesive strength, elongation at break, and contact contamination level of the adhesive embedded with PMMA/PEI nanoparticles were compared with those of conventional adhesive material.

## 2. Materials and Methods

### 2.1. Materials

The epoxy resin was bisphenol A type resin (YD-128, Kukdo Chemical, Seoul, Korea). Bisphenol A glycerolate dimethacrylate (BisGMA) as acrylate resin and fumed silica with a particle size of 0.25 µm and specific surface area of 395 m^2^ g^−1^ were purchased from Sigma-Aldrich (Sigma-Aldrich Korea, Seoul, Korea). Adipic acid dihydrazide (ADH) as conventional hardener was received from Tokyo Chemical Industry. Irgacure 651 (Ciba Specialty Chemicals, Basel, Switzerland) was used as a photoinitiator. To synthesize the PMMA/PEI core-shell nanoparticle, methyl methacrylate (MMA), branched PEI (M_n_ = 60,000), and *tert*-butyl hydroperoxide (TBHP; 70 wt% in H_2_O) were obtained from Sigma-Aldrich (Sigma-Aldrich Korea, Seoul, Korea).

### 2.2. Synthesis of Multifunctional PMMA/PEI Core-Shell Nanoparticle

PMMA/PEI core-shell nanoparticles were synthesized as described in a previous study [19]. 2.0 g of branched PEI was dissolved in 16.8 g of deionized water for 30 min under nitrogen purging. After 0.8 g of MMA and 256.0 μg of TBHP were added, the PMMA/PEI core-shell nanoparticles were reacted at 80 °C for 2 h under controlled agitation using a magnetic stir bar. The PMMA homopolymer was separated by filtration. Unreactive residues were purified by centrifugation, and then the synthesized PMMA/PEI nanoparticles were obtained by lyophilization.

### 2.3. Preparation of UV/Heat Dual-Curable Adhesives Embedded with PMMA/PEI Nanoparticles

The mixture of YD-128 epoxy resin, BisGMA acrylate resin, and fumed silica was prepared by using a rotation and revolution mixer (Thinky, AR-100, Tokyo, Japan) at 2200/1300 rpm for 15 min. Then, Irgacure 651 photoinitiator and hardeners involving conventional ADH or reactive PMMA/PEI core-shell nanoparticle were added to the mixture and blended twice at 2200/1300 rpm for 5 min. After mixing, the adhesive mixtures were defoamed in a vacuum oven at 25 °C for 2 h. As shown in Table 1, the contents of the synthesized PMMA/PEI nanoparticle were 0.5, 1.0, and 1.5 wt%.

### 2.4. Characterization

A field emission scanning electron microscopy (FE-SEM; Hitachi, SU-70, Tokyo, Japan) with an accelerating voltage of 15 kV was conducted to analyze the morphology and particle size of PMMA/PEI nanoparticles. Fourier transform infrared (FT-IR; Jasco, FT/IR-460 Plus, Easton, PA, USA) spectroscopy and differential scanning calorimetry (DSC; TA instrument, Q20, New Castle, DE, USA) were used for confirming the conversion ratio and glass transition temperature of the UV/heat dual-curable adhesive. An attenuated total reflectance accessory and KBr pellets were used for FT-IR measurements. The adhesive was heated from 50 °C to 200 °C at the rate 10 °C min^−1^ for DSC scanning. In order to confirm the conversion ratio, the curing condition for the adhesive was UV irradiation with an energy of 3.0 J cm^−2^ (Daihan Labtech, WUV-L50, Seoul, Korea), followed by heat-curing at 120 °C or 130 °C for 1 h. The narrow drawing experiment was conducted on Janome seal dispenser with pressure and drawing speed of 60 psi and 1 mm s^−1^, respectively.

In order to examine the adhesive strength, the UV/heat dual-curable adhesive was cured between two indium tin oxide (ITO) glass substrates. The two ITO glass substrates were assembled crosswise, and the diameter of the adhesive was 3 mm. Curing was performed similar to the process adopted for the conversion ratio analysis. The adhesive strength was measured using the pull-off method by a universal testing machine (UTM; LLOYD, LR-5K, Bognor Regis, UK) with a crosshead speed of 1.3 mm min^−1^. The elongation at the break of the adhesive was analyzed using lap shear method. The curing and evaluation procedure proceeded in the same manner as the measurement of adhesion strength.

To measure the contact contamination of the LCs, a test cell was fabricated using two ITO glass substrates, which were covered with a polyimide (PI) layer on the surface using a spin coater. The PI layer on the ITO glass substrate was soft-baked on a hotplate at 80 °C for 10 min and then hard-baked in an oven at 230 °C for 1 h. The adhesive was cured between PI-layer-coated ITO glass substrates according to the same curing condition as conversion ratio analysis, and then the LC was injected into the test cell. The fabricated LC test cells were examined by polarized optical microscopy (POM; Olympus, BX51, Tokyo, Japan) for detection of contact contamination of the LCs.

## 3. Results and Discussion

### 3.1. Synthesis of Multifunctional PMMA/PEI Core-shell Nanoparticles

**Figure 2:**
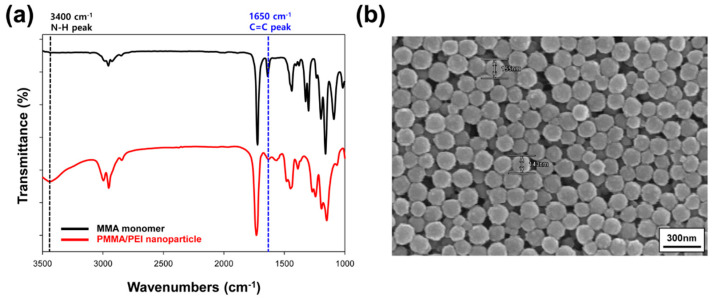
(**a**) FT-IR spectra of MMA monomer (black) and PMMA/PEI nanoparticle (red). (**b**) FE-SEM image of PMMA/PEI nanoparticle.

Reactive PMMA/PEI polymer nanoparticles were synthesized by graft copolymerization through one-pot method and were lyophilized after purification. Figure 2a shows the FT-IR spectra of MMA monomer and PMMA/PEI nanoparticle. The MMA monomer has a carbon–carbon double bond (C=C) peak at 1650 cm^−1^, which almost disappeared in the PMMA/PEI nanoparticles. The decrease in intensity of the carbon–carbon double bond peak for the PMMA/PEI nanoparticles is ascribed to the graft copolymerization of PMMA onto branched PEI chains [19]. In contrast, the stretching vibration corresponding to amine functional groups (-NH_2_) at 3400 cm^−1^, which is a characteristic peak for pure PEI [20], is not observed in MMA but can be detected in the PMMA/PEI nanoparticles, owing to the presence of PEI shells containing numerous amine groups that copolymerized with the PMMA core. The overall spectral features are in accord with the literature [20]. After synthesizing the multifunctional PMMA/PEI nanoparticles, their particle size and morphology were confirmed using a FE-SEM instrument. The PMMA/PEI nanoparticles exhibited the spherical shape with the average particle size of about 150 nm, as shown in Figure 2b.

### 3.2. Curing and Narrow Drawing Characteristics of New UV/Heat Dual-Curable Adhesives

**Figure 3:**
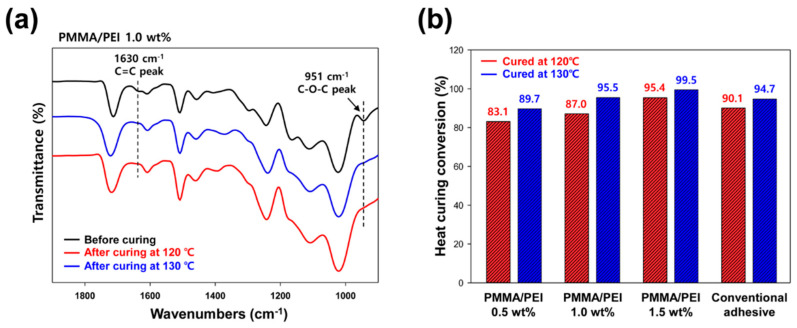
(**a**) FT-IR spectra of the UV/heat dual-curable adhesive with 1.0 wt% of PMMA/PEI nanoparticles before and after heat treatment at 120 or 130 °C. (**b**) Heat-curing ratios of conventional and new adhesives after heat treatment at 120 or 130 °C.

**Figure 4:**
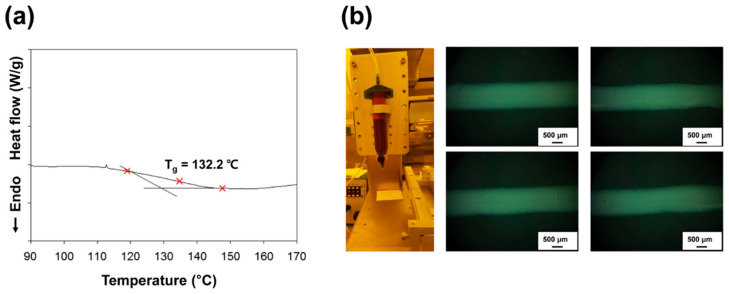
(**a**) DSC thermogram of the adhesive containing 1.5 wt% of PMMA/PEI nanoparticles. (**b**) Optical images of adhesive lines after drawing and sandwiching the adhesive between two glass substrates using a commercial seal dispenser.

The curing behavior of the UV/heat dual-curable adhesives was analyzed using FT-IR spectroscopy in the attenuated total reflection mode. Compared to conventional adhesive with ADH hardener, the proposed UV/heat dual-curable adhesive is embedded with reactive PMMA/PEI nanoparticles, containing a large number of amine functional groups that can react with the oxirane rings of the epoxy resin. In case of UV curing of acrylate groups, the peak area of C–C double bond at 1630 cm^−1^ was measured, and UV conversion ratio was calculated by obtaining the peak areas of acrylate group before and after UV treatment. High UV conversion of about 98% was obtained for the adhesive with 1.0 wt% PMMA/PEI nanoparticles irrespective of heat-curing temperature, as shown in Figure 3a. To examine the effect of heat-curing temperature on curing conversion of the adhesive, the peak area of C-O-C epoxide group at 951 cm^−1^ was analyzed, and the heat conversion ratio was calculated by Equation (1).

(1) $$\mathrm{Heat}\;\mathrm{conversion}\;\mathrm{ratio}\;\left(\%\right)=\frac{A_{pure}-A_{cured}}{A_{pure}}\times 100$$

where A_pure_ and A_cured_ are the peak area of epoxide group before and after heat treatment, respectively. A comparison of the results for the curing temperatures of 120 °C and 130 °C shows that a higher curing temperature has a higher heat conversion ratio, as shown in Figure 3b. The adhesive containing 1.0 wt% of PMMA/PEI nanoparticles cured at 120 °C has a curing ratio of 87.0%, but the one cured at 130 °C exhibits relatively high curing ratio of 95.5%. For confirming the difference in the heat-curing ratios, glass transition temperature of the adhesive was analyzed. Figure 4a shows the DSC thermogram of the proposed adhesive embedded with reactive PMMA/PEI nanoparticles, which confirms glass–rubber transition with a midpoint at 132.2 °C and an onset at 128.4 °C. Generally, heating a polymer above its T_g_ increases its mobility by loosening the links between the molecular chains. For this reason, heat treatment at 130 °C shows a better heat-curing ratio than at 120 °C because the curing temperature is close to the T_g_ of the proposed adhesive. Moreover, as the concentration of multifunctional PMMA/PEI nanoparticle increases, the heat-curing ratio increases, as shown in Figure 3b. Under the same curing temperature of 120 °C, a 0.5 wt% concentration of PMMA/PEI particles shows 83.1% heat-curing ratio, but 1.0 wt% and 1.5 wt% concentrations of PMMA/PEI particles exhibit 87.0% and 95.4% heat-curing ratios, respectively. In addition, when curing at 130 °C, the heat-curing ratio is 89.7%, 95.5%, and 99.5% for PMMA/PEI concentrations of 0.5, 1.0, and 1.5 wt%, respectively. Notably, new adhesive embedded with PMMA/PEI nanoparticles exhibits high heat-curing ratio at the same concentration of 1.5 wt% compared to that of conventional adhesive with ADH hardener, suggesting that multifunctional PMMA/PEI polymer nanoparticle can serve as efficient multi-curing agent for epoxy resin within the adhesive for display application. Since the synthesized PMMA/PEI nanoparticles have a large number of amine functional groups on the particle surface of branched PEI shell, these amine groups can act as hardeners to assist the curing reaction of the epoxy resin. Therefore, as the content of PMMA/PEI nanoparticles and the curing temperature increase, the adhesive exhibits a high degree of thermal curing ratio.

Figure 4b shows the optical images of adhesive lines after drawing and sandwiching the adhesive containing 1.5 wt% of PMMA/PEI nanoparticles between two glass substrates using a commercial seal dispenser. The uniform and straight seal lines with the narrow line width of about 1.2 mm are exhibited, indicating that the proposed adhesive embedded with polymer nanoparticles can afford excellent narrow drawing performance, which is essential for super-narrow bezel display due to the nano-sized characteristic of embedded PMMA/PEI nanoparticle.

### 3.3. Adhesive Strength and Elongation Property of New UV/Heat Dual-Curable Adhesives

**Figure 5:**
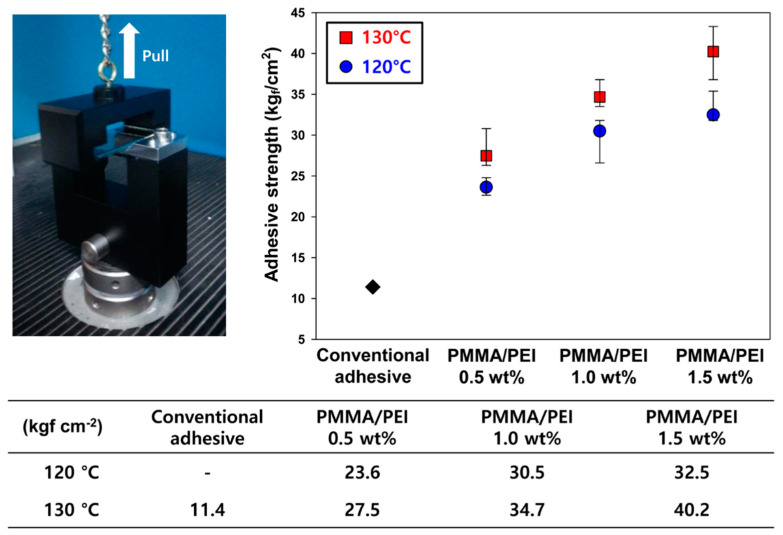
Adhesive strengths of conventional and new adhesives after heat treatment at 120 or 130 °C.

In order to investigate the effect of curing temperature and nanoparticle content on the mechanical properties of the adhesives embedded with PMMA/PEI nanoparticles, the adhesion strength of the proposed adhesive was compared with that of a conventional adhesive containing dihydrazide-type hardener. The pull-off test result of each adhesive is shown in Figure 5. In these results, it can be seen that the adhesive strength increases as the heat-curing temperature and concentration of PMMA/PEI nanoparticles increase. An adhesive containing 0.5 wt% PMMA/PEI cured at 120 °C shows a relatively low adhesive strength of 23.6 kgf cm^−2^. Since the curing temperature is below the T_g_ of adhesive resin and the PMMA/PEI concentration is low, the adhesive sample with 0.5 wt% nanoparticles has a weak crosslinked network compared to the other new adhesives. For the same curing temperature, when the PMMA/PEI nanoparticle concentration increases to 1.0 and 1.5 wt%, the adhesive strength also increases to 30.5 and 32.5 kgf cm^−2^, respectively. The increased PMMA/PEI concentration enhances the crosslinking sites between the epoxy resin and amine groups of the PMMA/PEI nanoparticles when it is cured under the same heat-curing temperature. Moreover, the adhesive strength obtained by heat-curing the adhesive at 130 °C is higher than that achieved by curing at 120 °C. The adhesion strength of the adhesive containing 0.5 wt% of PMMA/PEI cured at 130 °C is 27.5 kgf cm^−2^, which is higher than that of an adhesive with the same concentration cured at 120 °C. Moreover, when the concentration of PMMA/PEI nanoparticles increases to 1.0 and 1.5 wt%, the adhesive strength increases to 34.7 and 40.2 kgf cm^−2^, respectively. This result is attributed to the dual effect of high curing temperature, which is close to the T_g_ of the resin and the presence of numerous crosslinking sites between the epoxy resin and amine groups of nanoparticles [21,22,23]. Notably, the maximum adhesion strength of the new adhesive using the proposed PMMA/PEI nanoparticles was 352% higher than that of conventional adhesive.

**Figure 6:**
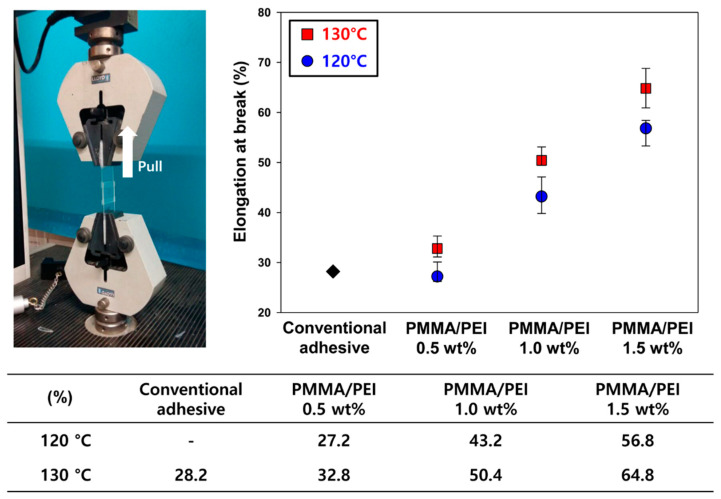
Elongations at break for conventional and new adhesives after heat treatment at 120 or 130 °C.

Figure 6 shows the results of the elongation at break for the conventional and new adhesives. The elongations of the adhesives containing the proposed core-shell nanoparticles were higher than that of conventional adhesive with ADH hardener, indicating that the new adhesives possess high toughness [24]. The adhesive cured at 130 °C has an average 20% higher elongation than the adhesive cured at 120 °C. With an increase in the heat-curing temperature and concentration of the PMMA/PEI nanoparticles, the density of the crosslinking network of nanoparticles increases due to high reaction mobility of adhesive resin and the increase in reaction site of the amine group, resulting in high toughness of the proposed adhesives.

### 3.4. Contact Contamination Characteristics of New UV/Heat Dual-Curable Adhesives

**Figure 7:**
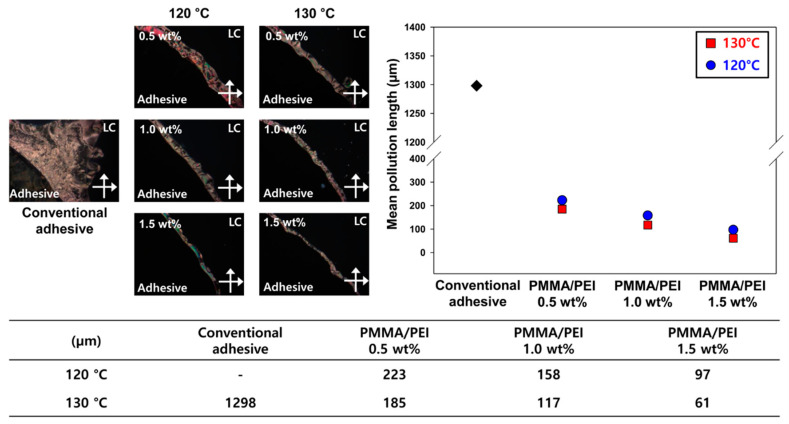
Mean pollution lengths of conventional and new adhesives after heat treatment at 120 or 130 °C.

During LC device fabrication, the adhesive and LCs are contacted directly in the LC injection process. When curing reaction of the adhesive is not complete, unreacted components of the adhesive can leak into the LC domain, which is the main reason for alignment defects in LC devices. When the alignment of the LC is disturbed by external stimuli, its orientation attains extinction, and Schlieren texture is formed due to random orientation of LCs. To judge the contact contamination level of LCs, the width of the Schlieren texture between adhesive and LC domains was measured using POM, and this width was named as the mean pollution length. Figure 7 shows the mean pollution lengths of the proposed adhesives and the conventional adhesive. The mean pollution length decreases with higher curing temperature and higher PMMA/PEI concentration. When cured at 120 °C, the mean pollution lengths for 0.5, 1.0, and 1.5 wt% were 223, 158, and 97 µm, respectively. Since the curing temperature of 120 °C exhibits relatively low curing conversion, the unreacted components can leak into the LC domain, leading to the increased pollution length. In contrast, the adhesive cured at 130 °C showed a lower mean pollution length than the adhesive cured at 120 °C. At 130 °C, when the concentration of PMMA/PEI nanoparticle increases from 0.5 to 1.5 wt%, the mean pollution length decreases from 185 to 61 µm. High curing temperature and high nanoparticle concentration induce an increase in the crosslinking sites, leading to a decrease in the amounts of unreacted components. Notably, the LC pollution length of the conventional adhesive was remarkably reduced by using the proposed PMMA/PEI nanoparticles, indicating the enhancement in reliability of the adhesive for display applications.

## 4. Conclusions

In this study, multifunctional PMMA/PEI core-shell nanoparticles were synthesized and employed as efficient multi-curing and toughening agents for highly adhesive and sustainable UV/heat dual-curable adhesives for super-narrow bezel display application. The characteristics of the proposed adhesives containing the PMMA/PEI nanoparticles were analyzed through measurements of curing conversion, narrow drawing ability, adhesive strength, elongation property, and contact contamination level of LCs. The results of curing conversion analysis confirmed that the PMMA/PEI nanoparticles could be used as an effective multi-curing agent for epoxy resins during the heat-curing process. In addition, it is found that the heat-curing ratio increases when the curing temperature and the content of PMMA/PEI nanoparticles increase due to high reaction mobility of epoxy resin near the glass transition temperature and the increase in the reaction site of the amine groups in nanoparticles. The drawing experiment using a commercial seal dispenser revealed that narrow and uniform seal lines can be achieved for the proposed adhesive due to the nano-sized characteristic of embedded PMMA/PEI core-shell nanoparticle. Based on the results for adhesive strength and elongation at break, it can be concluded that the heat-curing temperature and concentration of PMMA/PEI nanoparticles affect the mechanical properties of the proposed adhesives. With increases in the heat-curing ratio and nanoparticle concentration, the density of the crosslinking network of nanoparticles increases within the adhesive composite, leading to the improvement in adhesion strength and toughness of the proposed adhesive. Finally, the contact contamination of the LCs with the adhesives was measured for confirming its effect on the LC alignment, and it is found that as the curing conversion ratio increases, the amounts of unreacted components decrease, and thus the mean pollution length of LCs also decreases. A high heat-curing temperature along with high concentration of the PMMA/PEI nanoparticles can lead to the reduced pollution level between the LCs and adhesive material. Given that the proposed adhesive afforded excellent mechanical properties and outstanding reliability, the adhesive should find broad use in the fabrication of super-narrow bezel displays by offering a seamless characteristic and immersive environment.

## Figures and Tables

**Table 1:** Composition of conventional and new UV/heat dual-curable adhesives.

Composition (wt%)		Conventional Adhesive	New Adhesives Embedded with PMMA/PEI Nanoparticles		
Resin	YD-128	40	40	40	40
	BisGMA	40	40	40	40
Filler	Fumed silica	16	17	16.5	16
Hardener	ADH	1.5	-	-	-
	PMMA/PEI	-	0.5	1.0	1.5
Photoinitiator	Irgacure 651	2.5	2.5	2.5	2.5
